# Evaluating the Efficacy of Actinidia deliciosa (Kiwi Fruit) Extract in Inhibiting Pseudomonas aeruginosa Biofilm Formation: An In Vitro Study With Therapeutic Implications

**DOI:** 10.7759/cureus.70082

**Published:** 2024-09-24

**Authors:** Michael Zacharia Mathew, Abirami Arthanari, Shankar Ganesh, Naji Naseef Pathoor, Karthikeyan Ramalingam, Vignesh Ravindran

**Affiliations:** 1 Department of Forensic Odontology, Saveetha Dental College and Hospitals, Saveetha Institute of Medical and Technical Science (SIMATS) Saveetha University, Chennai, IND; 2 Department of Microbiology, Saveetha Dental College and Hospitals, Saveetha Institute of Medical and Technical Science (SIMATS) Saveetha University, Chennai, IND; 3 Department of Oral Pathology and Microbiology, Saveetha Dental College and Hospitals, Saveetha Institute of Medical and Technical Science (SIMATS) Saveetha University, Chennai, IND; 4 Department of Pediatric and Preventive Dentistry, Saveetha Dental College and Hospitals, Saveetha Institute of Medical and Technical Science (SIMATS) Saveetha University, Chennai, IND

**Keywords:** actinidia deliciosa, antibacterial, antibiofilm, anti-inflammatory, antioxidant, drug resistance, enzymes, gram negative, natural compounds, phytochemicals

## Abstract

Background

Gram-negative *Pseudomonas aeruginosa* is a common bacteria that is well-known for its capacity to build biofilms, which are organized cell communities encased in a self-produced polymeric matrix. Treating infections becomes more challenging due to biofilms' capacity to provide immunity and resistance to antibiotics. The search for novel anti-biofilm agents has gained significant momentum, and the diverse range of bioactive compounds found in natural products offers a promising avenue. Rich in vitamins, antioxidants, and various phytochemicals, *Actinidia deliciosa* (kiwi fruit) has demonstrated potential as an antibacterial agent.

Aim of the study

This study aims to assess the efficacy of *A. deliciosa *extract in inhibiting biofilm formation by *P. aeruginosa* in vitro, providing valuable insights into its potential as a natural therapeutic agent for preventing recurrent bacterial infections.

Materials and methodology

The antibacterial and antibiofilm properties of *A. deliciosa *(kiwi fruit) methanolic extract were assessed in this study against *P. aeruginosa *(PAO1). The fruit was gathered, examined by a botanist for authenticity, and then cold macerated in methanol to create an extract. A two-fold broth dilution procedure was used to calculate the minimum inhibitory concentration (MIC), and agar well-diffusion was used to evaluate the antimicrobial activity. Pyocyanin pigment quantification was carried out after the extract was applied, and the antibiofilm impact was evaluated using a crystal violet assay. GraphPad Prism (GraphPad Software, San Diego, CA) was used for statistical analysis.

Results

Based on our findings, *A. deliciosa* was shown to have significant antibacterial and antibiofilm properties against* P. aeruginosa* (PAO1). At 5 mg/mL, the methanolic extract of *A. deliciosa *exhibited antibacterial activity with an 8 mm zone of inhibition and suppressed PAO1 growth. At 2.5 mg/mL and 1.25 mg/mL doses, PAO1 significantly decreased the production of biofilms by 60% and 29%, respectively. In addition, pyocyanin pigment synthesis was decreased by 30% and 9.25%, respectively, at sub-MIC doses of 2.5 mg/mL and 1.25 mg/mL. When evaluated at 2.5 mg/mL, the extract did not, however, appreciably affect bacterial growth.

Conclusion

This study enhances the understanding of antibiotic resistance, alternative treatments, and pathogenic microbes. The in vitro findings suggest that A. deliciosa fruit extract may inhibit pyocyanin production in PAO1. Further research with different formulations is recommended to explore its anti-biofilm properties and potential pharmacological applications.

## Introduction

*Pseudomonas aeruginosa* is a rod-shaped bacterium classified as Gram-negative [1]. Pseudomonas infections have become increasingly prevalent in recent years, with more cases reported in the past decade compared to the previous century [2]. *P. aeruginosa* is a bacteria that is not very dangerous to healthy people, but it can be quite dangerous to those individuals who are immunocompromised or those who have other underlying medical conditions [1,2]. Patients with cystic fibrosis are particularly vulnerable to infections, but it also poses significant risks for individuals with chronic wounds, burns, obstructive pulmonary disease, and those with implanted biomaterials. Additionally, its presence on hospital surfaces and in water supplies can further endanger already at-risk patients [3]. *P. aeruginosa* bacterial species is a species that may change and adapt throughout infections [4]. Numerous studies have confirmed the highly infectious nature of the bacterium. Clinical strains were found to exhibit greater diversity compared to environmental isolates [5]. After a comprehensive investigation, it was discovered that water lines in dental offices provide an optimal environment for biofilm formation, which facilitates the multiplication and spread of the illness [6].

Intrinsic resistance describes a microbe's natural ability to withstand antimicrobial treatments. *P. aeruginosa*, an opportunistic pathogen, exhibits a high level of inherent resistance to numerous antimicrobial therapies [7,8]. Efflux pumps have been shown to confer resistance to a wide range of antimicrobials, including erythromycin, aminoglycosides, roxithromycin, chloramphenicol, and ketolides. The family of tripartite multidrug efflux system pumps helps *P. aeruginosa* establish both acquired and adaptive resistance by ejecting antimicrobial medicines and other harmful compounds from the cell in a proton-dependent way [9,10]. The MexXY-OprM pump contributes to *P. aeruginosa*'s natural resistance to gentamicin, erythromycin, and tetracycline [11,12]. This intrinsic resistance is further compounded by acquired resistance mechanisms, which affect various antibiotic classes, including aminoglycosides, fluoroquinolones, and β-lactams [13].

The kiwi fruit, *Actinidia deliciosa*, belongs to the family Actinidiaceae and the genus *Actinidia*. The literature states that there are 60 species of plants in the genus *Actinidia* [14]. The components of this tropical fruit are known to have therapeutic and medicinal properties that can be used to cure a variety of conditions, such as diabetes, cancer, digestive problems, renal disorders, and even cardiovascular diseases. Vitamin C is the most abundant phytochemical in kiwi fruit, and minerals are also abundant in it [15]. Kiwi fruit possesses antioxidant qualities and antibacterial activity. Apart from its antioxidant characteristics, kiwi fruit has exhibited antibacterial activity against certain pathogenic bacteria, such as *Escherichia coli*, *Listeria monocytogenes*, and *Staphylococcus aureus* [16]. Flavonoids and organic acids are two examples of the phenolic substances present in kiwi fruit. There are many phenolic chemicals found in the fruit's flesh, leaves, skin, and roots. Tri-terpenoids, which are distinguished by 12-en-28-oic acids of the ursane and oleanane kinds, are the primary compounds found in the roots of kiwi fruit. These crude extracts' bioactivities included antibacterial, anti-inflammatory, antioxidant, immunoregulatory, and anticancer effects. The well-known antibacterial, anti-inflammatory, and antioxidant properties of kiwi fruit extract help reduce inflammation and the symptoms and indicators of inflammatory diseases [17,18]. Our research seeks to advance natural product-based therapeutics by investigating the antibacterial potential of natural compounds, potentially leading to more effective antimicrobial medications and better disease management.

## Materials and methods

The study was conducted in the silver lab, and the study was approved by the ethical committee members with the number of SRB/SDC/FORENSIC-2210/24/216. This study was done in the presence of *A. deliciosa* samples, which were obtained from the local market in Chennai, Tamil Nadu, India. The fruit was first rinsed with distilled water, and then the fruit pulp samples were collected. Subsequently, the fruit was ground using an electronic grinder to achieve the necessary consistency for treatment against PAO1. Each experiment was conducted in triplicate, and both the growth curve analysis and biofilm quantification (using the crystal violet method) showed statistical significance. Data processing was performed using GraphPad Prism (GraphPad Software, San Diego, CA).

Solvent extraction, bacterial strain, and growth condition

For extracting the solvent, 20 g of the *A. deliciosa* pulp was subject to a cold maceration process in which the fruit sample was mixed in 100 mL of methanol and then distributed across two maceration containers for 48 hours. After the extraction phase, the resulting suspension was filtered using No. 1 filter paper (Whatman, Maidstone, England), which was layered over the funnel housing the filter paper with a white muslin cloth. A hot water bath precisely maintained at 50°C was used to concentrate the filtrate. After the dried material was carefully weighed and the desiccated filtrate was measured, it was stored at 4°C for future use. *P. aeruginosa* (PAO1) wild type was gifted by Dr. Busi Siddhardha, Pondicherry University, Puducherry. *P. aeruginosa* (PAO1) was sub-cultured in LB (Luria Bertani) (HiMedia, India) broth. The culture was kept for 24 hours at 37°C in a shaking incubator running at 100 rpm.

Antimicrobial activity of *A. deliciosa*

Agar well-diffusion, a standard technique, was used to assess *A. deliciosa* antibacterial activity. Using a sterile swab, the bacterial cultures with PAO1 tests were transferred to Mueller Hinton agar (MHA) plates (Hi Media, Mumbai, India). Ultimately, a sterile cork borer was used to puncture two 8-mm-diameter wells into the MHA medium. One well was filled with 40 μL of *A. deliciosa* extract, while the other well acted as a control. The plates were then incubated at 37°C in an upright position for the following 24 hours. After incubation, a Vernier caliper was used to measure the wells' zone in millimeters to discover *A. deliciosa's* antibacterial activity.

Antibiotic susceptibility testing and evaluation of the minimum inhibitory concentration (MIC)

The antimicrobial susceptibility against PAO1 was determined by employing the Kirby-Bauer disk diffusion method. This technique involves placing antibiotic-impregnated disks on an agar plate inoculated with PAO1 and then measuring the zones of inhibition around the disks to assess the effectiveness of the antibiotics. Using a sterile brush moistened with the bacterial suspension in opposition to conventional antibiotics, the inoculum containing the bacterial culture was distributed on the MHA plates. A two-fold broth dilution procedure assessed the MIC of *A. deliciosa *extract against PAO1 employed in the experiment. It was evaluated at various concentrations ranging from 10 mg/mL to 0.019 mg/mL. Using accepted protocols, the MIC of *A. deliciosa *against PAO1 was ascertained [19]. In summary, 20 μL of bacterial cultures with 0.5 McFarland turbidity standard units (1.5 x 108 CFU/mL) were added to tubes holding LB broth. Following each tube's dilution with *A. deliciosa*, they were all incubated for a day at 37°C. Following 24 hours, 40 μL of 2,3,5-triphenyl tetrazolium chloride (TTC) was introduced into the tubes, and any alterations in color were tracked and assessed. The lowest concentration at which no growth is observed is taken as the MIC. Based on these findings, the antibiofilm studies were conducted.

Crystal violet biofilm inhibition assay

An investigation using crystal violet staining was used to determine how *A. deliciosa* extract affected PAO1 biofilm formation [15]. A microtiter plate containing 180 µL of fresh LB medium was loaded with a 20 µL overnight culture of PAO1, and the extract was applied in a dose-dependent manner (5 mg/mL-0.009 mg/mL for PAO1). The sample was incubated at 37°C for 48 hours. After two days, surface-adherent biofilm was stained with a 0.1% crystal violet (CV) solution. After 15 minutes, the unbound CV was rinsed off with sterile distilled water. The CV bound to the adherent biofilm was then extracted using 200 µL of 70% ethanol, and the intensity of the crystal violet was measured at an optical density (OD) of 520 nm using a UV-vis spectrophotometer. To determine the percentage of growth inhibition, the OD at 600 nm of the treated strains was compared to that of the untreated control. The percentage of inhibition was calculated using the formula: ((Control OD 520 nm - Treated OD 520 nm) / Control OD 520 nm) × 100.

Bacterial growth curve and pyocyanin pigment quantification

*A. deliciosa* methanol extract at a concentration of 2.5 mg/mL was tested both with and without PAO1 bacterial growth. Then, for a maximum of 24 hours, the culture was incubated at 37°C, and the cell density was assessed at OD 600 nm every 60 minutes [19]. The overnight culture of PAO1 was used to extract pyocyanin method, and to summarize, a single colony of the PAO1 strain was cultured into nutrient broth tubes with varying concentrations of *A. deliciosa* methanol extract (ranging from 2.5 mg/mL to 0.019 mg/mL). Control tubes, on the other hand, were made without any *A. deliciosa* extract and were incubated for 24 hours at 37°C. Following incubation, a 10-minute, 6000-rpm centrifugation was performed on each tube. Both the treated (using *A. deliciosa*), and untreated PAO1 supernatants were centrifuged, and the resultant supernatants were then transferred to fresh tubes. Each tube was filled with chloroform, and the pyocyanin pigment found in the layer of chloroform was taken out and put in fresh tubes. The bright red solution likely contains a pH indicator that changes color in response to acidity. Then, the solution obtained was gently mixing 200 µL of 0.2 M HCl with this. Following a 10-minute centrifugation period at 8,000 rpm, 100 µL of the supernatant was moved to a microtiter plate in order to measure the OD at 520 nm. The percentage of growth of the treated strain of PAO1 within the pellet was measured by measuring the OD at 600 nm and comparing it to the control strain (which did not include *A. deliciosa* extract).

## Results

This study assessed the antimicrobial and anti-biofilm properties of *A. deliciosa* (kiwi fruit) against *P. aeruginosa* PAO1. The extract demonstrated antimicrobial activity, with an 8 mm zone of inhibition and significant biofilm reduction at sub-MIC concentrations of 2.5 mg/mL and 1.25 mg/mL, inhibiting biofilm formation by 60% and 29%, respectively. Although the extract did not affect bacterial growth at 2.5 mg/mL, it did reduce pyocyanin pigment production by 30% at the same concentration. Overall, *A. deliciosa *shows potential as a natural agent against PAO1 biofilms. The antibiotic susceptibility of *P. aeruginosa* PAO1. It is resistant (R) to azlocillin, aminoglycosides, piperacillin, and carbenicillin. However, it exhibits susceptibility to tobramycin, netillin, and amikacin, with inhibition zones of 20.3 ± 1.9 mm, 15 ± 0.9 mm, and 15.1 ± 0.5 mm, respectively (shown in Table 1).

**Table 1:** Analysis of antibiogram of Pseudomonas aeruginosa PAO1

S.no	Antibiotics	Pseudomonas aeruginosa PAO1 (mm)
1	Tobramycin	20.3 ± 1.9
2	Azlocillin	R
3	Aminoglycosides	R
4	Netillin	15 ± 0.9
5	Piperacillin	R
6	Amikacin	15.1 ± 0.5
7	Carbenicillin	R

*A. deliciosa* inhibits the growth of *P. aeruginosa *PAO1 at higher concentrations (10 mg/mL and 5 mg/mL), but the bacteria continue to grow at concentrations of 2.5 mg/mL and lower. This suggests that the antimicrobial effect of the extract diminishes as the concentration decreases (Table 2).

**Table 2:** Minimum inhibitory concentration of methanolic extract A. deliciosa against PAO1 “-” = Growth inhibited, “+” = Growth

S.no	Two-fold dilution concentration (mg/mL)	Growth measured PAO1
1	10	-
2	5	-
3	2.5	+
4	1.25	+
5	0.62	+
6	0.312	+
7	0.156	+
8	0.078	+
9	0.039	+
10	0.019	+

**Figure 1:**
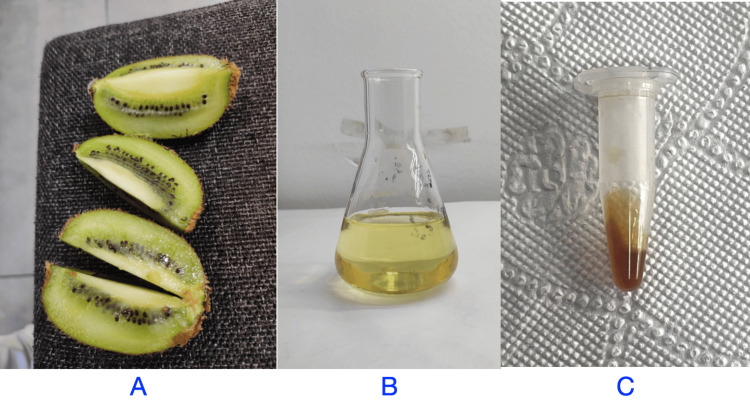
(A) A. deliciosa fruit. (B) The fruit was pulverized using an electronic grinder, following which it was subject to cold maceration. (C) Methanolic extract of A. deliciosa

The fruit of *A. deliciosa* was pulverized using an electronic grinder and then subjected to cold maceration to obtain a methanolic extract (Figure 1).

**Figure 2:**
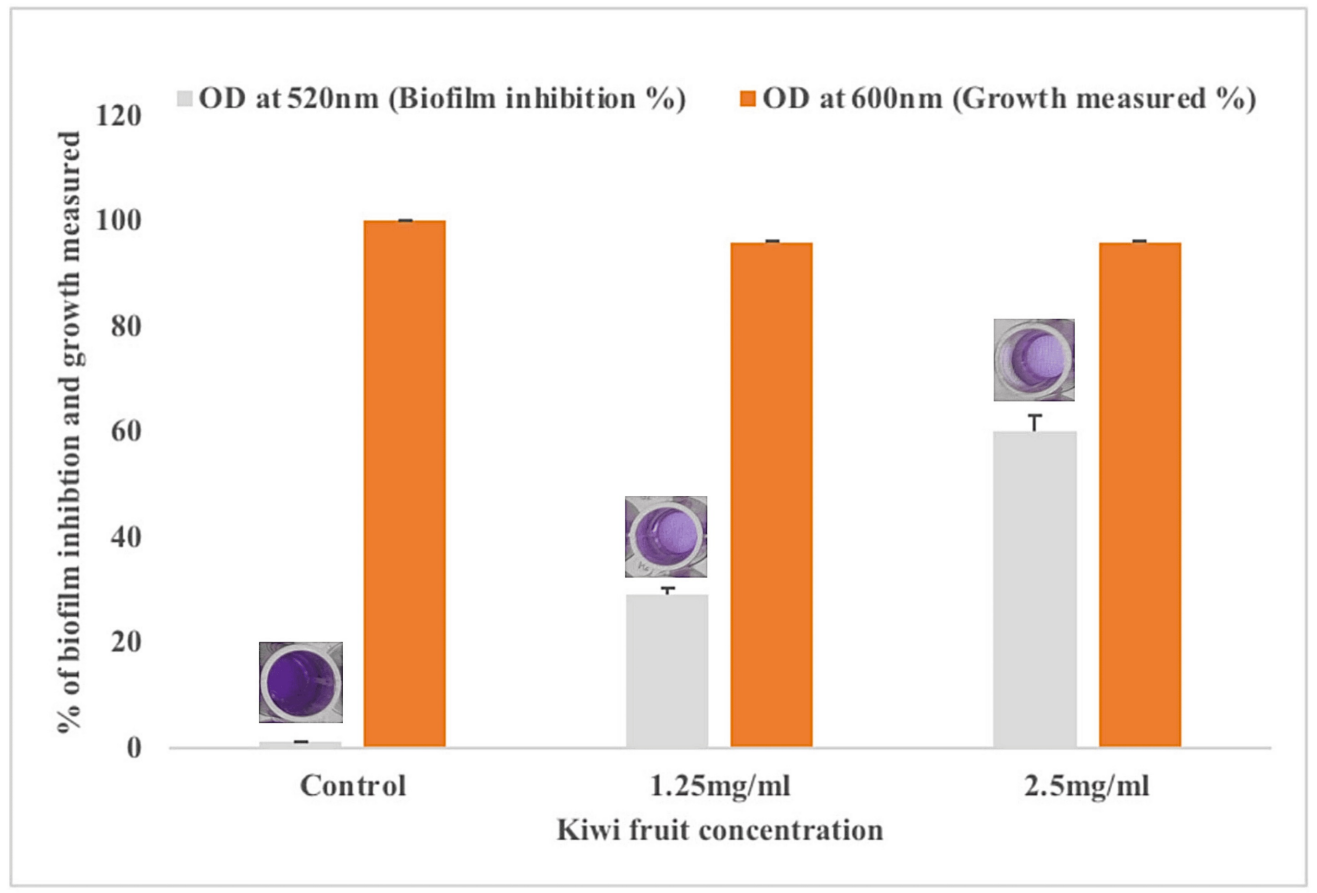
The methanol extract of A. deliciosa-inhibited PAO1 biofilm formation

Crystal violet biofilm inhibition assay. The methanol extract of *A. deliciosa* extract inhibited PAO1 biofilms at sub-inhibitory concentrations of 2.5 mg/mL and 1.25 mg/mL. Figure 2 shows 60% and 29% of biofilm inhibition in PAO1 at sub-inhibitory concentrations of 2.5 mg/mL and 1.25 mg/mL of *A. deliciosa *extract.

**Figure 3:**
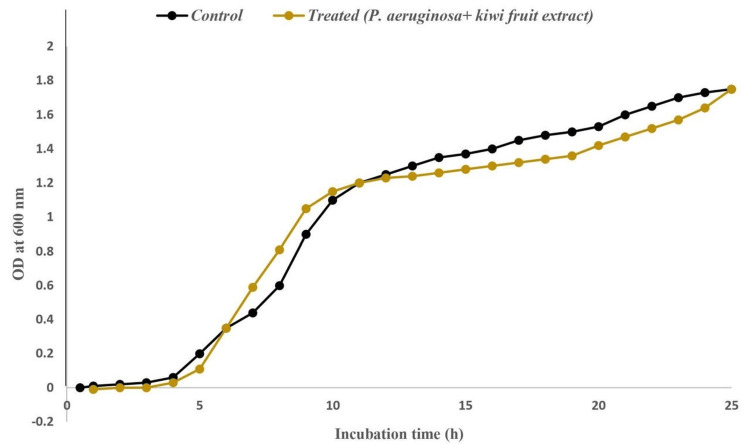
Growth curve analysis of A. deliciosa

Growth curve analysis was performed on PAO1 cultures. The control group was grown without *A. deliciosa* extract, while the experimental group was grown in the presence of *A. deliciosa* extract at a concentration of 2.5 mg/mL (Figure 3).

**Figure 4:**
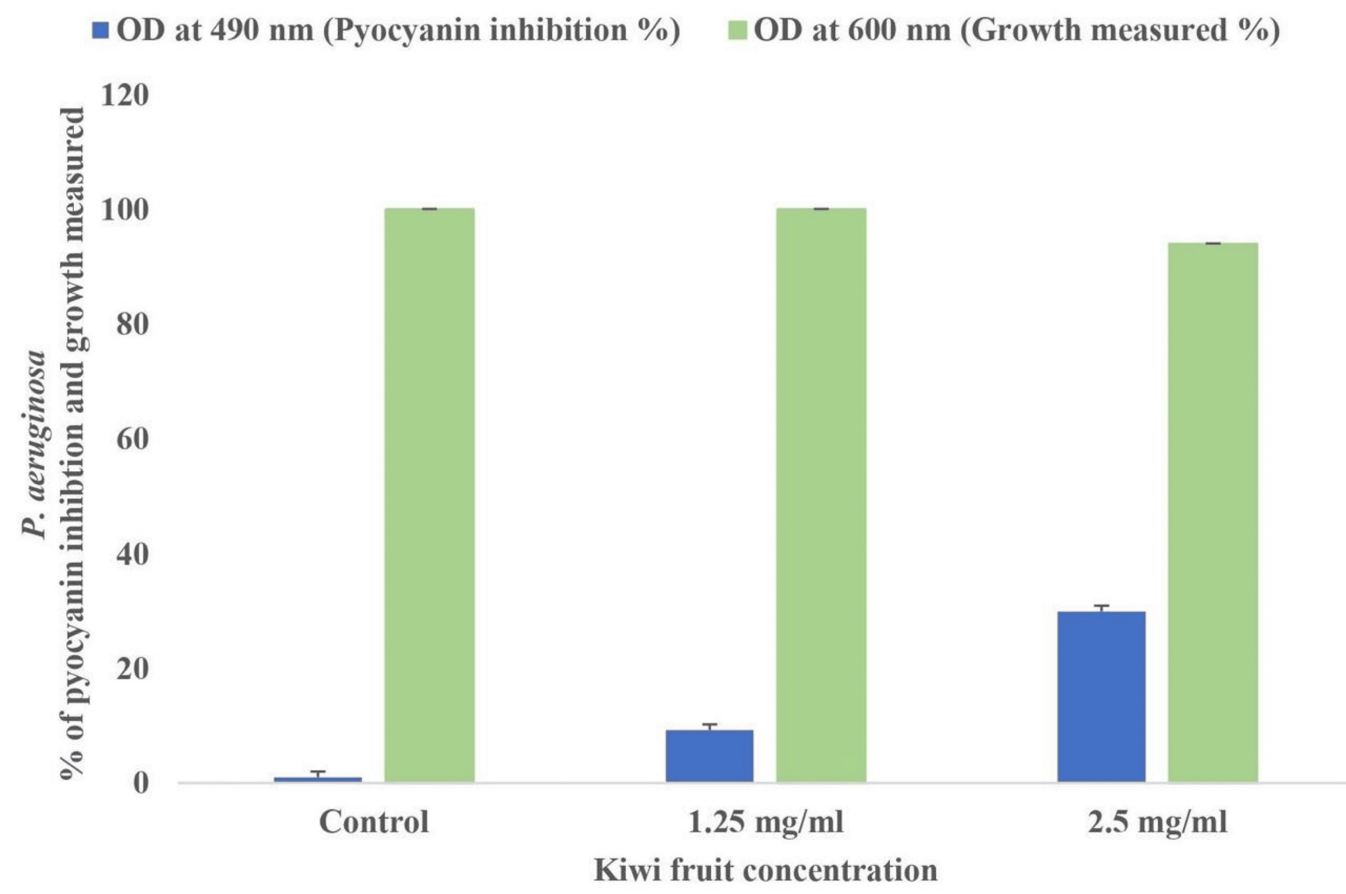
Graphical representation of pyocyanin inhibition and cell growth

The representation of pyocyanin inhibition and cell growth. Additionally, 30% and 9.25% of pyocyanin inhibition in PAO1 when treated with 2.5 mg/mL and 1.25 mg/mL of *A. deliciosa* extract, respectively, are shown in Figure 4.

## Discussion

The antibiotic susceptibility results of *P. aeruginosa* PAO1 indicate a pattern of resistance to several antibiotics, including azlocillin, aminoglycosides, piperacillin, and carbenicillin, while showing susceptibility to tobramycin, netillin, and amikacin, with notable inhibition zones. These findings suggest that PAO1 has developed mechanisms to evade certain antibiotics but remains vulnerable to specific treatments, which can guide clinical decisions. The methanol extract of *A. deliciosa *demonstrates a concentration-dependent inhibitory effect on the growth and biofilm formation of PAO1. At higher concentrations (10 mg/mL and 5 mg/mL), the extract effectively inhibits bacterial growth, while lower concentrations (2.5 mg/mL and below) allow for continued bacterial proliferation. Additionally, sub-inhibitory concentrations of 2.5 mg/mL and 1.25 mg/mL of the extract significantly reduce biofilm formation by 60% and 29%, respectively, and inhibit pyocyanin production by 30% and 9.25%. This suggests that *A. deliciosa *extract could be a potential adjunctive treatment to target biofilm-related infections and virulence factors in PAO1, particularly in the face of antibiotic resistance.

The opportunistic Gram-negative environmental species *P. aeruginosa* settles in immunocompromised and vulnerable patients, such as those with cystic fibrosis or those in intensive care units, and goes on to become a major global cause of nosocomial infections. Pigment synthesis and the manufacture of lytic enzymes are essential for *P. aeruginosa's* infectiousness. Antibiotic abuse and overuse have been selected for resistant types of bacteria, for which there are few effective treatment alternatives [20]. There are many factors that contribute to the virulence of *P. aeruginosa*. Out of them, we have concentrated on pyocyanin in this study. Our study concentrates on analyzing the involvement of pyocyanin in the biofilm-forming activity of *P. aeruginosa,* emphasizing the possibility of inhibiting these systems as a means of controlling infections. The importance of pyocyanin, its role in virulence, and its role in contributing toward molecular pathogenesis in relation to *P. aeruginosa* has been confirmed [21]. In our study, we have used a natural tropical fruit *A. deliciosa,* which has shown promising bioactive properties, in order to test its potential antimicrobial action. This study portrays the antibiofilm, as well as the antimicrobial action of *A. deliciosa* fruit extract against PAO1. On the primary examination and analysis, *A. deliciosa *fruit extract demonstrated the inhibition of bacterial activity at the lowest concentration of 5 mg/mL in PAO1. A study done by Nozohour et al. [22] showed that the tropical fruit, pomegranate, showed a significant amount of antibacterial activity against *P. aeruginosa* bacteria portraying an MIC value of 12.5 mg/mL.

The date fruit is a fruit that is commonly found in tropical regions in abundance. The recent investigations conducted by Al-Shwyeh et al. [23] suggest that the date fruit is rich in bioactive compounds that can exert an antioxidant action and thereby exhibit a significant and potent bactericidal action against *P. aeruginosa*. When looking into the aspect of utilizing leaves of plants for utilizing their medicinal properties in order to cure infections, especially those related to *P. aeruginosa*, a study conducted by Habbal et al. [24] showed that the henna leaves, which are commonly found in the tropical regions such as the Middle East and India, exhibited high in *P. aeuginosa* activity at a 50% concentration. In our study, we found out that *A. deliciosa* fruit extract inhibited pyocyanin-dependent biofilm formation of PAO1 in a dose-dependent manner at the lowest concentration of sub-MIC. In the crystal violet assay conducted, *A. deliciosa* fruit extract demonstrated a drastic lowering of the biofilm formation in PAO1 without affecting the planktonic cell development. The extract of kiwi fruit peel has been shown to exhibit cytotoxic action, as well as multi-drug resistance reversal in many resistant species such as *Staphylococcus epidermidis*, indicating and confirming its potential antimicrobial action [25]. The study conducted by Tiwari et al. [26] showed that the alkaloids and flavonoids present in *A. deliciosa* would be the main reason for its antibacterial activity. It was also seen that *A. deliciosa* exhibited a good amount of antibiofilm activity against *Acinetobacter baumannii. *Similarly, Rajkumari et al. [27] reported that, when the natural compound hordenine derived from *Hordeum vulgare* was used in the synthesis of gold nanoparticles, to be tested against *P. aeruginosa,* it showed a significant amount of *P. aeruginosa* biofilm inhibition up to 78.41%. Natural pyrrolidine alkaloid (R)-Bgugaine, which is isolated from *Arisarum vulgare*, is reported by Majik et al. [28] to reduce biofilm density by 83% and inhibit pyocyanin pigmentation and rhamnolipid synthesis. On the whole, it can be concluded that *A. deliciosa* could be crucial in inhibiting pyocyanin in PAO1. Additional studies that are more extensive are needed to isolate the exact bioactive compounds that confer the anti-biofilm activities.

Limitations

A limitation of this study is that it was conducted in vitro, which may not fully replicate the complex environment of a living organism. The biofilm inhibition observed in *P. aeruginosa* by kiwi fruit extract might differ under in vivo conditions due to factors such as immune response and bioavailability. Additionally, the specific concentration and preparation of the extract used in the study may not be directly translatable to practical therapeutic applications. Further research involving in vivo models is necessary to validate these findings.

## Conclusions

The results of the tests conducted were obtained, analyzed, and tabulated, and it can be concluded that this study adds significantly to our understanding of the intricate relationships between microorganisms that cause diseases, antibiotic resistance, and the potential of natural products to treat diseases. According to our findings, the natural *A. deliciosa* fruit extract has the potential to inhibit pyocyanin in PAO1. This potential is investigated through in vitro research. The pharmacological therapeutic applications of the discovered compound and its better understanding, particularly about their anti-biofilm capabilities, might be improved by conducting more extensive studies using various formulations.
